# Epigenetic Mechanisms of Endocrine-Disrupting Chemicals in Obesity

**DOI:** 10.3390/biomedicines9111716

**Published:** 2021-11-18

**Authors:** Immacolata Cristina Nettore, Fabiana Franchini, Giuseppe Palatucci, Paolo Emidio Macchia, Paola Ungaro

**Affiliations:** 1 Dipartimento di Medicina Clinica e Chirurgia, Università degli Studi di Napoli Federico II, Via S. Pansini, 80131 Naples, Italy; ic.nettore@gmail.com (I.C.N.); fa.franchini01@gmail.com (F.F.); gpalatucci21@gmail.com (G.P.); pmacchia@unina.it (P.E.M.); 2 Istituto per l’Endocrinologia e l’Oncologia Sperimentale del CNR “G. Salvatore”, Via S. Pansini, 80131 Naples, Italy

**Keywords:** epigenetic, endocrine-disrupting chemicals, obesity, obesogens, DNA methylation, histone modifications, microRNA

## Abstract

The incidence of obesity has dramatically increased over the last decades. Recently, there has been a growing interest in the possible association between the pandemics of obesity and some endocrine-disrupting chemicals (EDCs), termed “obesogens”. These are a heterogeneous group of exogenous compounds that can interfere in the endocrine regulation of energy metabolism and adipose tissue structure. Oral intake, inhalation, and dermal absorption represent the major sources of human exposure to these EDCs. Recently, epigenetic changes such as the methylation of cytosine residues on DNA, post-translational modification of histones, and microRNA expression have been considered to act as an intermediary between deleterious effects of EDCs and obesity development in susceptible individuals. Specifically, EDCs exposure during early-life development can detrimentally affect individuals via inducing epigenetic modifications that can permanently change the epigenome in the germline, enabling changes to be transmitted to the next generations and predisposing them to a multitude of diseases. The purpose of this review is to analyze the epigenetic alterations putatively induced by chemical exposures and their ability to interfere with the control of energy metabolism and adipose tissue regulation, resulting in imbalances in the control of body weight, which can lead to obesity.

## 1. Introduction

Adipose tissue represents the main storage of energy in the human body [1]. It is now recognized that adipose tissue function is regulated by endocrine system and that it behaves as an endocrine organ producing hormones. Therefore, inappropriate deposits of fat could be derived from interference in the hormonal control of adipose tissue functions and be themselves responsible for endocrine dysregulations [2].

Obesity is a metabolic condition characterized by the expansion of fat mass due to the increase in size (hypertrophy) and number (hyperplasia) of adipocytes [1]. Since 1980, the prevalence of obesity has more than doubled globally, and therefore, this condition is a serious threat to human health [3]. Nowadays, the incidence of obesity reaches about 600 million adults, and around 40 million children under the age of five years are overweight or obese. Worryingly, 80% of these children remain obese in adulthood [4]. Obesity is associated with and contributes to the development of several chronic disorders, including diabetes mellitus, metabolic syndrome, cardiovascular diseases, carcinogenesis, and infertility [3,5,6].

Dysregulation of endocrine and metabolic systems associated with environmental factors and genetic heritability represent the main causes of the widespread pandemic of obesity [7]. Exposure to certain environmental pollutants, especially present in wealthy industrialized countries, leads to an alteration of the endocrine system. Most of these pollutants are molecular analogues of natural estrogens and are classified as endocrine-disrupting chemicals (EDCs). These compounds, having a high affinity for estrogenic and androgenic receptors (ERs), modulate the endocrine pathways and can contribute to the global increase of diseases including obesity, diabetes, neurological disorders, and cancers [8,9].

Recently, EDCs captured scientists’ attention in consideration of their ability to modulate the epigenetic status of exposed individuals [10,11,12].

The epigenome can be considered a link between individual genetic background and the environment and determines the ability of an organism to response and adapt to environmental changes [13]. Individual phenotype is determined by both genotype and epigenotype. Their interaction with aberrant environmental conditions may produce modifications in gene expression. Diet and other environmental conditions influence epigenetic plasticity that, together with genetic modifications, is associated with the development of common complex diseases [13,14,15,16,17,18].

Endocrine disruptors can alter the epigenetic pathways, and, especially in children, they induce epigenetic modifications during development, responsible for an increased susceptibility to obesity. Therefore, the knowledge of epigenetic events induced by these substances is fundamental to define the role of EDCs in health assessment and onset of obesity.

## 2. Epigenetics and Obesity

**Figure 1:**
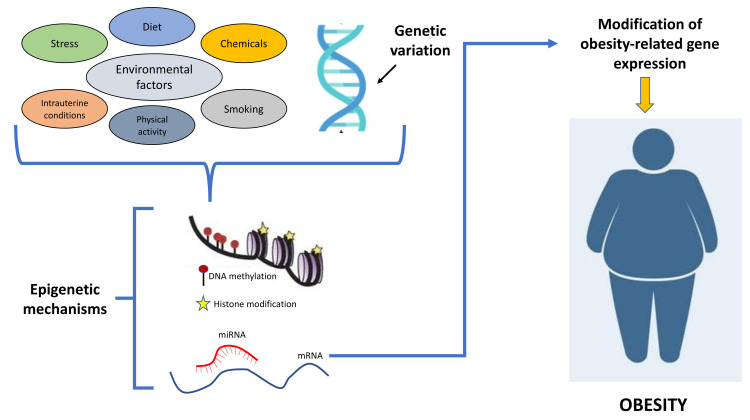
Schematic representation showing how the interplays between environmental factors and genetic variation contribute to obesity development through epigenetic mechanisms.

Epigenetic mechanisms regulate gene expression through reversible heritable changes that are transmitted across generations and that do not alter DNA sequence. The most studied epigenetic processes controlling gene expression through changes of chromatin architecture and binding of transcription factors are the methylation of cytosines within CpG dinucleotides, post-translational modifications of histones (i.e., acetylation/deacetylation or methylation/demethylation of lysine residues) catalyzed by histone-modifying enzymes [1], and microRNAs regulating multiple mRNAs [19,20,21,22,23,24,25]. Environmental factors, including EDCs, may be involved in obesity development by altering the epigenetic mechanisms [26,27,28] (Figure 1).

Several studies have investigated the association between DNA methylation and obesity in human tissue. The largest published epigenome-wide association study involved 5465 individuals and identified 37 CpG sites associated with body mass index [12]. These methylation sites are located within different genes involved in several metabolic processes, such as HIF3A, CPT1A, ABCG1, and SREBF1 genes [29]. HIF3A encodes for the hypoxia-inducible factor 3 subunit alpha, regulating responses to low oxygen (hypoxia) [16]; CPT1A encodes the enzyme carnitine palmitoyltransferase 1A, playing a role in carnitine-dependent transport across the mitochondrial membrane; ABCG1 is involved in macrophage cholesterol and phospholipids transport; SREBF1 encodes for a transcription factor associated with lipid metabolism [30]. Other CpG sites were identified in several genes whose methylation status was associated with body mass index (BMI) and waist circumference; these CpG sites are located in the body of the suppressor of cytokine signaling 3 (*SOCS3*) gene, in the 3**′** untranslated region of the zinc finger protein 771 (*ZNF771*) gene, and in the transcription start site of the LIM domain-containing 2 (*LIMD2*) gene [31].

Transcriptional analyses also showed associations between histone-modifying enzymes and obesity [1]. Indeed, expression of some histone deacetylases (HDACs), a class of enzymes regulating adipocyte differentiation and metabolism, has been found reduced in the adipose tissue of obese women [32]. Overnutrition in rodents and humans influences the expression of SiRT1, an enzyme belonging to the class III HDACs implicated in adipogenesis regulation. In obese patients, it has been observed that circulating levels of sirtuins are strongly modulated by environmental factors such as diet [33]. Various HDAC inhibitors have been produced based on different HDACs activities. These HDAC inhibitors are generally used to treat some forms of cancer. Indeed, it has been reported that down-regulation of HDAC3 decreases tumor proliferation [34]. In obesity, inactivation of HDAC3 increases or decreases the expression of different genes related to lipidogenesis and fatty acid and lipid oxidation. For instance, a HDAC3-selective inhibitor, RGFP966, is capable of activating fatty acid oxidation genes in the small intestine of C57B2/6WT mice [34]. Therefore, selective HDAC3 inhibitors might represent a potential therapeutic strategy in the treatment of obesity. Moreover, histone demethylases, another group of histone-modifying enzymes involved in adipocyte differentiation [1], have been linked to the development of obesity-associated inflammation [35]. In certain mouse models of obesity, a decreased expression of the histone demethylase LSD1 was found [36]. This enzyme is responsible for promoting brown fat differentiation, leading to a decrease in fat deposition and prevention of an obese phenotype [1].

MicroRNAs (miRNAs), small molecules that sequester the mRNA for degradation and/or prevent its translation by interfering with translation machinery, are also involved in both adipose tissue expandability and insulin resistance. For example, in adipose tissue from humans with low birthweight, increased levels of miR-483 and inhibition of the growth differentiation factor 3 (GDF-3) have been found. As consequence, the expandability of adipose tissue is reduced and ectopic fat deposition, which is one of the main causes determining insulin resistance, is increased [37].

Another illustrative example is miR-33. This miRNA regulates both glucose and lipid metabolism. Mice with reduced miR-33 expression are characterized by an abnormal food intake, causing obesity and insulin resistance [37]. By contrast, elevated expression of miR-128-1 is involved in human obesity. Indeed, miR-128-1 is broadly expressed in human adipose tissue, muscle, and liver and regulates the expression of genes encoding PPAR transcription factors and other modulators involved in fatty acid oxidation, energy expenditure, and inflammation [38]. Mice with a reduced expression of miR-128-1 fed with a calorie-rich diet are characterized by increased insulin sensitivity derived from reduced weight gain and less fat accumulation [38]. Several other miRNAs have been involved in the onset of obesity. Among these, miR-19, miR-29, miR-103, miR-107 and miR-451 have all been found expressed in obese mice [39,40,41,42].

## 3. Epigenetic Changes Induced by Obesogenic EDCs Exposure

**Figure 2:**
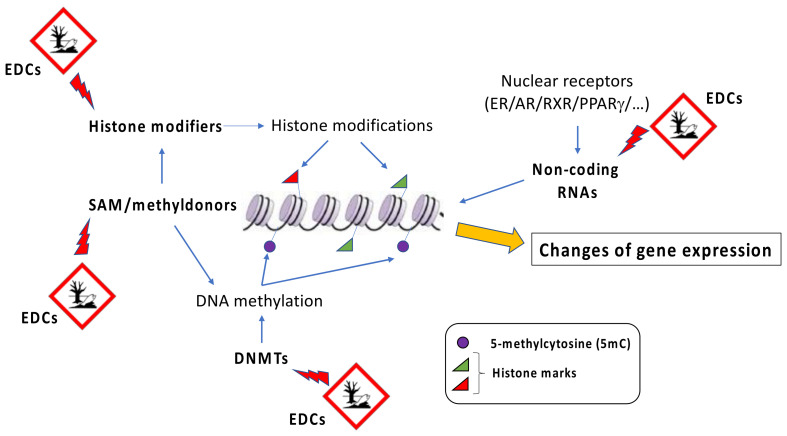
EDCs-induced epigenetic alterations. EDCs exposure may alter the levels of DNA methyltransferase (DNMT), histone modifiers, and SAM, as well as non-coding RNAs. All these events modify DNA methylation patterns and chromatin state and expression at specific genomic loci, determining changes in gene expression. SAM = s-adenosylmethionine.

EDCs are considered a heterogeneous group of natural (e.g., plant phytoestrogens) or synthetic compounds (e.g., industrial solvents, plastics, heavy metals, pesticides/herbicides) that cause health problems in an intact organism and its progeny by changing endocrine function. By binding to hormone receptors, EDCs influence downstream patterns regulated by specific hormones and lead to an imbalance in metabolism [43,44]. Dioxin and dioxin-like compounds, plastic components such as bisphenol A (BPA) and phthalates, parabens, and various flame retardants represent different examples of EDCs. Although heterogeneous, EDCs display some general characteristics shared by different components of the group. For instance, EDCs promote their adverse effects even at very low doses of exposure, and their action is stronger if it takes place during critical developmental periods, such as fetal life, infancy, puberty, and pregnancy [45,46,47]. In addition, the onset of disease promoted by EDCs is evident many years after the exposure [48]. Although EDCs show a very low affinity for hormone receptors compared to natural ligands, they can cause profound damage in several tissues. In general, since different classes of EDCs are released by human activities into the environment, they all together contribute to the development of a disease, and it is difficult to predict the detrimental effect associated with a specific EDC [49]. A possible association between the increase of global obesity and the spread of industrial chemicals into the environment was initially proposed in the early 2000s [50]. Afterwards, Grun and Blumberg introduced the term “obesogens” to indicate “xenobiotic chemicals that can disrupt the normal developmental and homeostatic controls over adipogenesis and/or energy balance” [51]. The effects of obesogenic environmental pollutants on adipose tissue expansion are stronger if the exposure happens during the prenatal or early-life period [52]. Obesogens target transcription regulators that are involved in the control of lipid homeostasis as well as adipocytes’ proliferation and differentiation. Due to their lipophilic property, EDCs accumulate in the adipose tissue over the years [53,54]. This produces a continuous spiral, promoting the EDC-induced expansion of adipose tissue and therefore the possibility to store larger amounts of additional EDCs [19]. Several obesogenic EDCs are known to affect the activity of a group of nuclear hormone receptors known as peroxisome proliferator-activated receptors (PPARs). Among those, PPARγ is considered the master regulator of adipogenesis, and it is associated with the control of lipids and glucose metabolism [55,56,57]. PPARγ is therefore the main target of obesogenic EDCs. In addition, since PPARs heterodimerize with the retinoid X receptors (RXRs) to induce transcription, RXRs can also be a target of obesogenic EDCs [58,59]. Although epigenetic effects of EDCs have been well described, the exact mechanisms by which they interfere with epigenetic marks still remain unknown. In general, it has been proposed as a global action, where EDCs affect the abundance or the activity of epigenetic regulators, such as DNA methyltransferases, and/or their cofactors, such as methyl donor SAM (s-adenosylmethionine), or they could have a gene-specific action, influencing the regulation of locus-specific epigenetic patterns (Figure 2).

Below is presented a description of the main EDCs more commonly associated with obesity and the epigenetic changes caused by exposures to them that are responsible for the development of obesity and other related disorders (Table 1).

### 3.1. Bisphenol A

Bisphenol-A (BPA) is an organic synthetic compound largely used in the manufacture of polycarbonate plastics and epoxy resins, two components used in many consumer products, including food containers, baby bottles, medical devices, and the lining of food cans [81]. BPA is one of the most produced chemicals worldwide; indeed, it has been estimated that the production of BPA each year reaches about 6 million tons [82]. Foods and water can be contaminated by BPA monomers because of its leaching from the plastic containers. BPA has been associated with many diseases such as diabetes mellitus, obesity, polycystic ovarian disease, cardiovascular disease, thyroid, reproductive and neurodevelopmental disorders, and cancers [83,84,85,86].

BPA interacts with several ERs, including ERα and ERβ, and these interactions regulate the expression of estrogen-responsive genes [87,88,89]. The relative binding affinity of BPA for these receptors is much lower than that of estradiol, although it binds with high specificity and a binding affinity constant (KD) of 5.5–5.7 nM to ERRgamma. This receptor represents the most recently identified member of the estrogen-related receptor (ERR) family, and it is present in the developing embryo and neonate. Therefore, it could be responsible for some for the effects of BPA during development [90]. Since both pancreatic islets and adipocytes express functional ERs [91], they become targets of BPA, which can induce insulin resistance, alteration in pancreatic beta-cell function, hepatotoxicity, and obesity [61]. Urinary BPA concentration in man has been associated with a high incidence of obesity [92]. In addition, a positive correlation between BPA urinary levels and insulin resistance was found in obese children regardless of BMI. This could be linked to the effect of BPA on the expression of adiponectin and resistin genes, as demonstrated in adipocyte cultures [93]. In these cells, BPA is capable of reducing adiponectin production and secretion and induce resistin expression, a condition that generally characterizes obese subjects, where adiponectin levels are usually reduced and resistin levels are elevated, determining insulin resistance [94].

Experiments conducted in 3T3-L1 cells (mouse fibroblast cells that can differentiate into adipocytes) and human adipose stromal/stem cells have demonstrated that BPA promotes the expression of PPARγ and c/EBPα, the two master regulators of adipogenesis, increases triglyceride content, and inhibits adiponectin release [61]. Globally, these effects stimulate adipogenesis. Interestingly, the adipogenic effects of BPA are mediated not only by estrogen receptors, but also by the influence on the activity of other enzymes, including 11beta-hydroxysteroid dehydrogenase type 1 [95], thyroid receptor/retinoid X receptor, or mammalian target of rapamycin signaling pathways [96]. BPA may induce changes in DNA methylation and determine histone modifications [60]. In vitro studies conducted in 3T3-L1 cells exposed to BPA confirm that this compound decreases global DNA methylation and enhances adipocyte differentiation [60]. This study demonstrates that altered epigenetic gene regulation may play a role in the link between BPA exposure and obesity development. In male rats, early-life exposure to BPA is responsible for increased fat/lean mass and adulthood hepatic steatosis with reduced mitochondrial function. These alterations are accompanied by changes in the epigenetic regulation of genes involved in hepatic beta-oxidation, such as the carnitine palmitoyltransferase (*Cpt1a*) gene. Here, BPA exposure promotes the binding of several transcription factors induced by modifications in DNA methylation and histone marks, such as histones H3 and H4 acetylation (H3Ac, H4Ac), histone di-methylation on lysine 4 on histone H3 (H3Me2K4), and histone tri-methylation on lysine 36 on histone H3 (H3Me3K36). Therefore, BPA toxicity is determined by both DNA methylation and histone modifications [62].

### 3.2. Diethylstilbestrol

Diethylstilbestrol (DES) is a synthetic non-steroidal estrogen that was prescribed between 1940 and 1971 to pregnant women to prevent adverse pregnancy outcomes [97]. Studies conducted in animals have suggested that exposure to DES during the prenatal or perinatal period increased the susceptibility to develop obesity during growth [63]. One possible explanation is that early exposure to DES may alter the genetic and epigenetic programming of adipocytes and their distribution.

In mice, an increase has been reported in circulating levels of leptin and pro-inflammatory cytokines, such as interleukin 6 (IL-6), considered markers of adiposity, during the early phase of exposure to DES. In addition, DES exposure has also been linked to alterations in glucose metabolism accompanied by pancreatic beta-cell hyperplasia [63,64]. DES exposure may potentially determine epigenetic effects, although epigenetic mechanisms that associate DES with obesity are still unclear. Recent studies demonstrated that many long non-coding RNAs (lncRNAs) regulate adipogenesis [98] and lipid homeostasis [99] and are regulated by nutrient factors and metabolic hormones [100]. Interestingly, several cancer-related lncRNAs are dysregulated/co-expressed in obesity, suggesting that obesity-associated lncRNAs may promote cancers. Therefore, data associating miRNA induction by EDCs and obesity-induced cancers could represent a link between EDCs exposure, miRNA expression, and obesity onset. As an example, in a breast cancer cell line (MCF-7), Bhan and coauthors have demonstrated that BPA and DES induce the expression of oncogenic long non-coding RNA HOTAIR (HOX transcript antisense RNA) [65], a potential oncogene having a significant impact on tumor cell viability, proliferation, and invasion [100,101]. A recent study demonstrated that a sedentary lifestyle further increases circulating exosomal HOTAIR in obese subjects, but not in lean subjects [43]. Ectopic expression of HOTAIR in abdominal preadipocytes produced an increase in the differentiation and expression of key adipogenic genes including *PPARγ* and *LPL* (lipoprotein lipase), the main enzyme of lipid storage in adipocytes [66]. Thus, one possibility is that a large increase in HOTAIR expression as a consequence of BPA and DES exposure may drive an increase in the differentiation in abdominal preadipocytes determining regional adiposity. 

### 3.3. Phthalates

Phthalates are diesters of phthalic acid and are used to improve the flexibility, transparency, and durability of plastic materials, such as polyvinyl chloride (PVC). For this reason, they are present in many consumer products, including children’s toys, food and beverage packaging, and medical devices. Human exposure to phthalates is generally due to dermal contact with PVC and plastic materials that release phthalates or by inhalation or ingestion [102].

Mice models have shown that phthalates’ metabolites represent one of the causes of the increasing incidence of metabolic disease and that a close correlation between phthalates, increased adipogenesis, and insulin resistance exists [67]. Indeed, during adipocyte differentiation, phthalates activate PPARγ receptors [68]. A recent study has demonstrated a correlation between urinary excretion of phthalates’ metabolites and obesity in both males and females, and phthalates exposure in children increases the risk of obesity [69]. These effects can be determined by the anti-androgenic actions of these compounds, which low cause androgenic activity and are responsible for the development of overweight and obesity [103,104]. DNA methylation represents a potential mechanism by which phthalate exposure in utero may exert long-term effects. An example is the study conducted by Miura and collaborators on DNA methylation in the cord blood of 203 mother–child pairs after di-2-ethylhexyl phthalate (DEHP) exposure. The results obtained have identified increased methylation changes associated with prenatal DEHP exposure at the level of genes related to metabolism, the endocrine system, and signal transduction. Further, increased methylation changes associated with DEHP exposure may contribute to the effects of prenatal exposure to this chemical on fetal growth [67].

Among the phthalates, butyl benzyl phthalate (BBP) is ubiquitously present in multiple products and can enter cells, bioaccumulate, and lead to extensive exposure to humans. Studies showed that 3T3-L1 preadipocytes exposed to BBP were induced to differentiate into mature adipocytes [67,105,106] and were characterized by the induction of miR-34a-5p expression, a key miRNA involved in obesity. In parallel, a decrease in the expression levels of *Nampt* and *Sirt1*, two target genes of miR-34a-5p, is observed, along with another significant epigenetic regulator, Sirt3 [67]. Zhang and collaborators have demonstrated that BBP exposure may regulate insulin signaling by altering vital epigenetic regulators, such as long noncoding RNA H19, and their downstream pathways [70].

### 3.4. Organochlorine and Organophosphate Pesticides

Organochlorine pesticides (OCPs) are chlorinated hydrocarbons that were used from 1940 to 1960. Although banned in several countries, some of these compounds tend to persist in the environment and bioaccumulate [71], posing a serious risk to worldwide human health. OCPs have been substituted by organophosphates (OPPs) that are esters of phosphoric acid and are commonly used as insecticide [72]. The first largely used compound of this group and one of the mostly known is dichlorodiphenyltrichloroethane, or DDT. Due to their high lipophilicity, these compounds become stored in fatty tissue and may act as endocrine disrupters [71]. Their presence in human adipose tissue has been linked to obesity and insulin resistance, probably due to their interference in *PPARγ* gene expression, production of inflammatory cytokines, such as tumor necrosis factor-α (TNF-α), and anti-androgenic effect [73]. A recent study demonstrated that a breakdown product of DDT, p,p’-dichlorodiphenyldichloroethylene (DDE), enhances adipogenesis and intracellular lipid accumulation in 3T3-L1 cells through the up-regulation of some proteins involved in lipid storage, such as fatty acid-binding protein 4 and sterol regulatory element-binding protein-1c [107]. Early-life exposure to OPPs has been associated with hyperinsulinemia and hyperlipidemia, characteristic features of a prediabetic condition. In addition, human ApoE-targeted replacement mice showed an increase in food intake and weight gain after chronic dietary exposure to chlorpyrifos, one of the most frequently used OPPs worldwide. This suggested that genetic factors may also modulate the response to toxic exposure to OPPs and the susceptibility to the development of obesity and other related metabolic dysfunctions [108].

Pesticides can be used as an example of how exposure to EDCs during development causes alterations in epigenetic gene regulation, leading to adipogenic or obesity-related effects. Studies in 3T3-L1 preadipocytes have demonstrated that tributyltin chloride (TBT) exposure is associated with a global DNA reduced methylation level, promoting adipocyte differentiation [60]. This phenomenon has also been observed in adipose-derived stromal cells (ADSCs) isolated from mice exposed in utero to TBT. These cells presented an increased trend to differentiate into adipocytes rather than osteocytes [74]. ADSCs are characterized by a demethylation in the promoter region of some PPARγ target genes, such as *Fapb4*. These cells are also characterized by an enhancement in PPARγ levels that is not accompanied by changes in DNA methylation levels. This increase could be due to a demethylation of lysine 27 on histone H3 (H3K27me3) after pesticides exposure [75].

### 3.5. Inhaled Pollutants

Toxic environmental particles represent a worldwide public health problem. They originate from a variety of sources, including industrial sources, automobile traffic, and natural disasters, such as volcanic eruptions and forest fires [76,77]. Generally, the classification of air pollutants is based on the source of their origin, their chemical composition, and the mode and space of their release that could be gaseous or particulate and indoor or outdoor. Regardless of their origin, these pollutants are a mixture of gases and particulate matter (PM) with toxic effects [109]. Data from large epidemiological studies have indicated an association between air pollution exposure and cardiovascular morbidity and mortality [110], as well as increased lung cancer risk [111,112]. Moreover, a meta-analysis study indicated that PM2.5 susceptibility to cardiovascular diseases is strongly influenced by obesity, suggesting that obese people show a higher risk of developing cardiovascular disease after exposure to inhaled pollution particles. Moreover, long-term exposure to PM enhances the expression of local pro-inflammatory mediators that translocate from the lung into the circulation, leading to an increase of the classic systemic inflammatory response that paves the way for the onset of obesity, type 2 diabetes, insulin resistance, and metabolic syndrome [78].

An association between PM2.5 exposure and nonalcoholic fatty liver disease (NAFLD) has also been described, and it is due to the potential of the pollutants to promote cytokines secretion from the Kupffer cells in the liver [56]. Moreover, systemic inflammation induced by inhaled pollutants activates the hypothalamic–pituitary–adrenal (HPA) axis that in turn inhibits somatotropic, thyrotropic, and gonadal axes, all exerting relevant effects on body composition and weight gain [113,114]. Thus, the dysregulation of the HPA axis caused by inhaled pollutants might cause such endocrine perturbations that impact body composition, leading to obesity and non-transmissible chronic disease onset.

Polycyclic aromatic hydrocarbons (PAHs), a family of air pollutants generated during incomplete combustion with both carcinogenic and endocrine-disrupting properties, may also influence obesity development. These substances bind to DNA and might alter the methylation state of PPARγ and PPARγ target genes, therefore acting as “obesogens” [79].

### 3.6. Flame Retardants

Flame retardants, such as polybrominated diphenyl ethers (PBDEs) and polybrominated biphenyls (PBBs), are a group of EDCs generally added to manufactured materials, such as plastics, textiles, and surface finishes and coatings to prevent or slow the further development of ignition. A positive association between serum PBDEs and body mass index was found in several studies [115,116,117,118].

An example of how these EDCs can produce epigenetic effects associated with obesity development is given by the flame retardant BDE-47. This compound is responsible for a dose-dependent adipocyte differentiation and a reduction in global DNA methylation levels [60], as demonstrated in the *PPARγ2* gene promoter. Moreover, exposure to BDE-47 generally leads to the increased expression of different adipogenic genes, including leptin gene (*LEP*), although their expression levels are not linked to changes in DNA methylation [80].

## 4. Epigenetic Inheritance Determined by Obesogenic EDCs

Different environmental toxicants, including the fungicide vinclozolin [119,120], plastics (bisphenol A and phthalates) [121], pesticide (diethylmetatoluamide and permethrin) [122], dioxin [123], hydrocarbons [124], and DTT [125], are reported to be responsible for the epigenetic inheritance of adult-onset disease in future generation progeny. The mechanisms of this non-genetic form of inheritance are based on germline transmission of epigenetic information across generations [119,120]. The alterations in the germline epigenome caused by an environmental insult escape the epigenetic reprogramming that happens after fertilization. As consequence, all derived cell types will have an altered epigenome and transcriptome determining the susceptibility to develop adult-onset disease across generations [126,127].

Recently, a prospective study has been published that was conducted on a children’s cohort at ages 2, 4, 6, and 8 years to evaluate the relationship between CpG methylation status and BMI in relation to maternal exposure to BPA. The study demonstrated that there was an increase in the methylation levels associated with the IGF2R gene at age 2, but not at age 6, in the group whose mothers presented high BPA urinary levels during pregnancy. Changes in IGF2R methylation levels were associated with increased BMI during ages 2–6 in girls, but not in boys, suggesting that a possible sensitive period of DNA methylation occurs during development and that BPA exposure, by modulating the methylation status, may influence BMI during development in a sex-specific manner [128]. Manikkam et al. reported that the pesticide methoxychlor, an approved insecticide and pesticide replacing DDT in agricultural application, is capable of inducing DNA methylation changes in the sperm of the F3 generation of gestating rats. These differentially methylated regions (DMR), called epimutations, are found to be specific to methoxychlor exposure and are associated to an increased incidence of obesity and kidney and ovary diseases [129]. In mice, the offspring of dams exposed to elevated PAHs levels during gestation presented with increased weight and fat mass as well as higher gene expression of PPARγ, C/EBPα, Cox2, FAS, and adiponectin and lower DNA methylation in the PPARγ promoter. Similar differences in phenotype and DNA methylation extended through the grand-offspring mice [79].

These data indicate that prenatal environmental exposures may induce increased weight, fat mass, adipose gene expression, and epigenetic changes not only in the progeny, but also through the grand-offspring generations (F2, F3).

## 5. Discussion and Conclusions

Although the field of environmental obesogens is still young, the studies included in this review indicate that exposure to EDCs may determine epigenetic modifications and underlie the development of obesity and obesity-related diseases during the entire life. Adipocytes are strongly influenced by EDCs exposure, being themselves endocrine cells producing and receiving endocrine signals from different endocrine tissues. The obesogen effects of EDCs are determined by different elements, including genetic, epigenetic, and environmental factors, such as parental dietary habits and lifestyle. The mechanisms determining the obesogen effects of EDCs early in life could be different since they can be modulated by the action of individual (chronic) exposure to EDCs throughout the entire life span. Moreover, the epigenetic alterations produced by exposure to EDCs can also be transmitted to future generations.

Up to now, in the context of obesogenic EDCs and epigenetic modifications, the general picture of the interplays between DNA methylation, histone modifications, and noncoding RNA is still uncompleted, with most studies being focused on DNA methylation. Early-life EDCs exposure can alter epigenetic programming of obesity by activating or inhibiting nuclear receptors and other transcription factors, which in turn recruit chromatin-modifying complexes, such as methyl- and acetyltransferases, which regulate the expression of the target genes by directly altering epigenetic marks [130]. As described before, the principal research of obesogenic EDCs has been focused on PPARγ, considered the main target of obesogens. In particular, some EDCs, including TBT, BDE-47, and PAHs, bind to PPARγ and determine the alteration of its methylation status and of its target genes. Now, other mechanisms of EDC action have been discovered. As an example, BPA can induce the activation of ERα, which binds to estrogen-responsive elements present in the promoter of the histone tri-methyltransferase EZH2 and recruits other coregulators, such as histone acetyltransferases and methyltransferases. These proteins create a permissive state of chromatin and expression of EZH2 protein that trimethylates target genes, potentially affecting global epigenetic gene regulation [131,132]. Other studies have demonstrated that EDCs exposure can also regulate the expression and enzymatic activity of DNA methyltransferases and affect the level of their cofactors such as the methyl donor SAM, which is involved in several methylation reactions [133].

Further epidemiological studies linking EDCs exposure, epigenetic gene modifications, and obesity are needed in humans to better understand the effects of developmental exposure to EDCs and identify epigenetic biomarkers related to adult-onset obesity. DNA methylation can be considered a useful target for examining the effects of developmental exposure to EDCs, providing relevant information on the relationships between EDCs exposure and health. As an example, promoter methylation of retinoid X receptor in umbilical cord tissue is linked to 26% of the variation in childhood adiposity [134] and could therefore be used as target for evaluating the effects of EDCs exposure.

In conclusion, additional studies focused on the discovery of epigenetic biomarkers through omics technologies could enable key fundamental knowledge to understand the effects of developmental exposure to EDCs and latent onset of obesity in humans.

## Figures and Tables

**Table 1:** Characteristics and epigenetic effects of the main obesogenic EDCs.

Endocrine Disruptor	Description	Obesogenic Actions	Epigenetic Effects	Refs.
Bisphenol A (BPA)	Synthetic organic compound used in polycarbonate and resins. Commonly detected in water bottles, food containers, and metal-based cans.	Stimulation of adipogenesis Induction of insulin resistance Alteration of pancreatic beta-cell function Hepatotoxicity Induction of adulthood hepatic steatosis Reduction in mitochondrial function	Reduction in global DNA methylation Changes in histone marks (H3Ac, H4Ac, H3K4me2, H3K36me3)	[60] [61] [62]
Diethylstilbestrol (DES)	Synthetic estrogen used to prevent adverse pregnancy outcomes.	Stimulation of markers of adiposity (leptin and proinflammatory cytokines, [IL-6]) Alteration in glucose metabolism and pancreatic beta-cell hyperplasia	Increased expression of long non-coding RNA HOTAIR.	[63] [64] [65] [66]
Phthalates	Diesters of phthalic acid, widely used in the production of plastic products (children’s toys, food packaging, medical devices, and furnishings).	Increased adipogenesis and insulin resistance Strong correlation between urinary levels of phthalates’ metabolites and obesity	Increased DNA methylation at level of genes related to metabolism Increased expression of miR-34a-5p and of long non-coding RNA H19 and its downstream pathway	[67] [68] [69] [67] [70]
Organochlorine (OCPs) and Organophosphate (OPPs) Pesticides	OCPs are chlorinated hydrocarbons used from the 1940s to the 1960s and are still detected in tap water. OPPs represent up to 50% of all the insecticide use worldwide.	Stimulation of adipogenesis OPPs accumulate in adipose tissue and influence PPARγ gene expression and production of inflammatory cytokines	Decreased global DNA methylation in both adipose-derived stromal cells (ADSCs) and 3T3-L1 preadipocytes Increased demethylation of lysine 27 on histone H3 (H3K27me3)	[71] [72] [73] [60] [74] [75]
Inhaled pollutants	Toxic environmental particles originate from a variety of sources (industrial pollution, automobile traffic, natural disasters).	Stimulation of the classic systemic inflammatory response associated with obesity, type 2 diabetes, insulin resistance, and metabolic syndrome	Altered DNA methylation status of PPARγ and PPARγ target genes	[76] [77] [78] [79]
Flame retardants	Group of compounds that prevent or slow the further development of ignition.	Stimulation of adipocytes differentiation Strong association between polybrominated diphenyl ethers (PBDEs) exposure and body mass index	Reduction in global DNA methylation	[80]

## References

[1] Macchia P.E., Nettore I.C., Franchini F., Santana-Viera L., Ungaro P. (2021). Epigenetic regulation of adipogenesis by histone-modifying enzymes. Epigenomics.

[2] Hampton T. (2006). Scientists study fat as endocrine organ. JAMA.

[3] Vassilopoulou L., Psycharakis C., Petrakis D., Tsiaoussis J., Tsatsakis A.M. (2017). Obesity, Persistent Organic Pollutants and Related Health Problems. Adv. Exp. Med. Biol..

[4] Simmonds M., Llewellyn A., Owen C.G., Woolacott N. (2016). Predicting adult obesity from childhood obesity: A systematic review and meta-analysis. Obes. Rev..

[5] Perfetti A., Oriente F., Iovino S., Alberobello A.T., Barbagallo A.P., Esposito I., Fiory F., Teperino R., Ungaro P., Miele C. (2007). Phorbol esters induce intracellular accumulation of the anti-apoptotic protein PED/PEA-15 by preventing ubiquitinylation and proteasomal degradation. J. Biol. Chem..

[6] Willinger L., Brudy L., Meyer M., Oberhoffer-Fritz R., Ewert P., Muller J. (2021). Overweight and Obesity in Patients with Congenital Heart Disease: A Systematic Review. Int. J. Environ. Res. Public Health.

[7] Swinburn B., Egger G. (2002). Preventive strategies against weight gain and obesity. Obes. Rev..

[8] Kabir E.R., Rahman M.S., Rahman I. (2015). A review on endocrine disruptors and their possible impacts on human health. Environ. Toxicol. Pharmacol..

[9] Nettore I.C., Colao A., Macchia P.E. (2018). Nutritional and Environmental Factors in Thyroid Carcinogenesis. Int. J. Environ. Res. Public Health.

[10] Pigeyre M., Yazdi F.T., Kaur Y., Meyre D. (2016). Recent progress in genetics, epigenetics and metagenomics unveils the pathophysiology of human obesity. Clin. Sci..

[11] Ollikainen M., Ismail K., Gervin K., Kyllonen A., Hakkarainen A., Lundbom J., Jarvinen E.A., Harris J.R., Lundbom N., Rissanen A. (2015). Genome-wide blood DNA methylation alterations at regulatory elements and heterochromatic regions in monozygotic twins discordant for obesity and liver fat. Clin. Epigenetics.

[12] Demerath E.W., Guan W., Grove M.L., Aslibekyan S., Mendelson M., Zhou Y.H., Hedman A.K., Sandling J.K., Li L.A., Irvin M.R. (2015). Epigenome-wide association study (EWAS) of BMI, BMI change and waist circumference in African American adults identifies multiple replicated loci. Hum. Mol. Genet..

[13] Russo G.L., Vastolo V., Ciccarelli M., Albano L., Macchia P.E., Ungaro P. (2017). Dietary polyphenols and chromatin remodeling. Crit. Rev. Food Sci. Nutr..

[14] Petronis A. (2010). Epigenetics as a unifying principle in the aetiology of complex traits and diseases. Nature.

[15] Nettore I.C., Rocca C., Mancino G., Albano L., Amelio D., Grande F., Puoci F., Pasqua T., Desiderio S., Mazza R. (2019). Quercetin and its derivative Q2 modulate chromatin dynamics in adipogenesis and Q2 prevents obesity and metabolic disorders in rats. J. Nutr. Biochem..

[16] Lima R.S., de Assis Silva Gomes J., Moreira P.R. (2020). An overview about DNA methylation in childhood obesity: Characteristics of the studies and main findings. J. Cell. Biochem..

[17] Raciti G.A., Longo M., Parrillo L., Ciccarelli M., Mirra P., Ungaro P., Formisano P., Miele C., Beguinot F. (2015). Understanding type 2 diabetes: From genetics to epigenetics. Acta Diabetol..

[18] Porta M., Amione C., Barutta F., Fornengo P., Merlo S., Gruden G., Albano L., Ciccarelli M., Ungaro P., Durazzo M. (2019). The co-activator-associated arginine methyltransferase 1 (CARM1) gene is overexpressed in type 2 diabetes. Endocrine.

[19] Kim J.K., Samaranayake M., Pradhan S. (2009). Epigenetic mechanisms in mammals. Cell. Mol. Life Sci..

[20] Jones P.A., Takai D. (2001). The role of DNA methylation in mammalian epigenetics. Science.

[21] Reik W. (2007). Stability and flexibility of epigenetic gene regulation in mammalian development. Nature.

[22] Castel S.E., Martienssen R.A. (2013). RNA interference in the nucleus: Roles for small RNAs in transcription, epigenetics and beyond. Nat. Rev. Genet..

[23] Petryk N., Bultmann S., Bartke T., Defossez P.A. (2021). Staying true to yourself: Mechanisms of DNA methylation maintenance in mammals. Nucleic Acids Res..

[24] Cavalieri V. (2021). The Expanding Constellation of Histone Post-Translational Modifications in the Epigenetic Landscape. Genes.

[25] Dexheimer P.J., Cochella L. (2020). MicroRNAs: From Mechanism to Organism. Front. Cell Dev. Biol..

[26] Capparelli R., Iannelli D. (2021). Role of Epigenetics in Type 2 Diabetes and Obesity. Biomedicines.

[27] Vastolo V., Nettore I.C., Ciccarelli M., Albano L., Raciti G.A., Longo M., Beguinot F., Ungaro P. (2018). High-fat diet unveils an enhancer element at the Ped/Pea-15 gene responsible for epigenetic memory in skeletal muscle. Metabolism.

[28] Ciccarelli M., Vastolo V., Albano L., Lecce M., Cabaro S., Liotti A., Longo M., Oriente F., Russo G.L., Macchia P.E. (2016). Glucose-induced expression of the homeotic transcription factor Prep1 is associated with histone post-translational modifications in skeletal muscle. Diabetologia.

[29] Ling C., Ronn T. (2019). Epigenetics in Human Obesity and Type 2 Diabetes. Cell Metab..

[30] Jones A.C., Irvin M.R., Claas S.A., Arnett D.K. (2021). Lipid Phenotypes and DNA Methylation: A Review of the Literature. Curr. Atheroscler. Rep..

[31] Ali O., Cerjak D., Kent J.W., James R., Blangero J., Carless M.A., Zhang Y. (2016). Methylation of SOCS3 is inversely associated with metabolic syndrome in an epigenome-wide association study of obesity. Epigenetics.

[32] Jannat Ali Pour N., Meshkani R., Toolabi K., Mohassel Azadi S., Zand S., Emamgholipour S. (2020). Adipose tissue mRNA expression of HDAC1, HDAC3 and HDAC9 in obese women in relation to obesity indices and insulin resistance. Mol. Biol. Rep..

[33] Chang H.C., Guarente L. (2014). SIRT1 and other sirtuins in metabolism. Trends Endocrinol. Metab..

[34] Adhikari N., Jha T., Ghosh B. (2021). Dissecting Histone Deacetylase 3 in Multiple Disease Conditions: Selective Inhibition as a Promising Therapeutic Strategy. J. Med. Chem..

[35] Hanzu F.A., Musri M.M., Sanchez-Herrero A., Claret M., Esteban Y., Kaliman P., Gomis R., Parrizas M. (2013). Histone demethylase KDM1A represses inflammatory gene expression in preadipocytes. Obesity.

[36] Duteil D., Tosic M., Lausecker F., Nenseth H.Z., Muller J.M., Urban S., Willmann D., Petroll K., Messaddeq N., Arrigoni L. (2016). Lsd1 Ablation Triggers Metabolic Reprogramming of Brown Adipose Tissue. Cell Rep..

[37] Ferland-McCollough D., Fernandez-Twinn D.S., Cannell I.G., David H., Warner M., Vaag A.A., Bork-Jensen J., Brons C., Gant T.W., Willis A.E. (2012). Programming of adipose tissue miR-483-3p and GDF-3 expression by maternal diet in type 2 diabetes. Cell Death Differ..

[38] Wang L., Sinnott-Armstrong N., Wagschal A., Wark A.R., Camporez J.P., Perry R.J., Ji F., Sohn Y., Oh J., Wu S. (2020). A MicroRNA Linking Human Positive Selection and Metabolic Disorders. Cell.

[39] Trajkovski M., Hausser J., Soutschek J., Bhat B., Akin A., Zavolan M., Heim M.H., Stoffel M. (2011). MicroRNAs 103 and 107 regulate insulin sensitivity. Nature.

[40] Dou L., Meng X., Sui X., Wang S., Shen T., Huang X., Guo J., Fang W., Man Y., Xi J. (2015). MiR-19a regulates PTEN expression to mediate glycogen synthesis in hepatocytes. Sci. Rep..

[41] Liang J., Liu C., Qiao A., Cui Y., Zhang H., Cui A., Zhang S., Yang Y., Xiao X., Chen Y. (2013). MicroRNA-29a-c decrease fasting blood glucose levels by negatively regulating hepatic gluconeogenesis. J. Hepatol..

[42] Zhuo S., Yang M., Zhao Y., Chen X., Zhang F., Li N., Yao P., Zhu T., Mei H., Wang S. (2016). MicroRNA-451 Negatively Regulates Hepatic Glucose Production and Glucose Homeostasis by Targeting Glycerol Kinase-Mediated Gluconeogenesis. Diabetes.

[43] Le Magueresse-Battistoni B., Labaronne E., Vidal H., Naville D. (2017). Endocrine disrupting chemicals in mixture and obesity, diabetes and related metabolic disorders. World J. Biol. Chem..

[44] Vandenberg L.N., Colborn T., Hayes T.B., Heindel J.J., Jacobs D.R., Lee D.H., Shioda T., Soto A.M., vom Saal F.S., Welshons W.V. (2012). Hormones and endocrine-disrupting chemicals: Low-dose effects and nonmonotonic dose responses. Endocr. Rev..

[45] Rhomberg L.R., Goodman J.E. (2012). Low-dose effects and nonmonotonic dose-responses of endocrine disrupting chemicals: Has the case been made?. Regul. Toxicol. Pharmacol..

[46] Tabb M.M., Blumberg B. (2006). New modes of action for endocrine-disrupting chemicals. Mol. Endocrinol..

[47] Schug T.T., Janesick A., Blumberg B., Heindel J.J. (2011). Endocrine disrupting chemicals and disease susceptibility. J. Steroid Biochem. Mol. Biol..

[48] Diamanti-Kandarakis E., Bourguignon J.P., Giudice L.C., Hauser R., Prins G.S., Soto A.M., Zoeller R.T., Gore A.C. (2009). Endocrine-disrupting chemicals: An Endocrine Society scientific statement. Endocr. Rev..

[49] Ribeiro E., Ladeira C., Viegas S. (2017). EDCs Mixtures: A Stealthy Hazard for Human Health?. Toxics.

[50] Baillie-Hamilton P.F. (2002). Chemical toxins: A hypothesis to explain the global obesity epidemic. J. Altern. Complement. Med..

[51] Grun F., Blumberg B. (2006). Environmental obesogens: Organotins and endocrine disruption via nuclear receptor signaling. Endocrinology.

[52] Gore A.C., Chappell V.A., Fenton S.E., Flaws J.A., Nadal A., Prins G.S., Toppari J., Zoeller R.T. (2015). EDC-2: The Endocrine Society’s Second Scientific Statement on Endocrine-Disrupting Chemicals. Endocr. Rev..

[53] Cheikh Rouhou M., Karelis A.D., St-Pierre D.H., Lamontagne L. (2016). Adverse effects of weight loss: Are persistent organic pollutants a potential culprit?. Diabetes Metab..

[54] Ho S.M., Johnson A., Tarapore P., Janakiram V., Zhang X., Leung Y.K. (2012). Environmental epigenetics and its implication on disease risk and health outcomes. ILAR J..

[55] Anghel S.I., Wahli W. (2007). Fat poetry: A kingdom for PPAR gamma. Cell Res..

[56] Christodoulides C., Vidal-Puig A. (2010). PPARs and adipocyte function. Mol. Cell. Endocrinol..

[57] Ungaro P., Mirra P., Oriente F., Nigro C., Ciccarelli M., Vastolo V., Longo M., Perruolo G., Spinelli R., Formisano P. (2012). Peroxisome proliferator-activated receptor-gamma activation enhances insulin-stimulated glucose disposal by reducing ped/pea-15 gene expression in skeletal muscle cells: Evidence for involvement of activator protein-1. J. Biol. Chem..

[58] Grun F., Blumberg B. (2009). Endocrine disrupters as obesogens. Mol. Cell. Endocrinol..

[59] Grun F., Blumberg B. (2009). Minireview: The case for obesogens. Mol. Endocrinol..

[60] Bastos Sales L., Kamstra J.H., Cenijn P.H., van Rijt L.S., Hamers T., Legler J. (2013). Effects of endocrine disrupting chemicals on in vitro global DNA methylation and adipocyte differentiation. Toxicol. In Vitro.

[61] Chevalier N., Fenichel P. (2015). Endocrine disruptors: New players in the pathophysiology of type 2 diabetes?. Diabetes Metab..

[62] Strakovsky R.S., Wang H., Engeseth N.J., Flaws J.A., Helferich W.G., Pan Y.X., Lezmi S. (2015). Developmental bisphenol A (BPA) exposure leads to sex-specific modification of hepatic gene expression and epigenome at birth that may exacerbate high-fat diet-induced hepatic steatosis. Toxicol. Appl. Pharmacol..

[63] Newbold R.R., Padilla-Banks E., Snyder R.J., Phillips T.M., Jefferson W.N. (2007). Developmental exposure to endocrine disruptors and the obesity epidemic. Reprod. Toxicol..

[64] Newbold R.R. (2011). Developmental exposure to endocrine-disrupting chemicals programs for reproductive tract alterations and obesity later in life. Am. J. Clin. Nutr..

[65] Bhan A., Hussain I., Ansari K.I., Bobzean S.A., Perrotti L.I., Mandal S.S. (2014). Bisphenol-A and diethylstilbestrol exposure induces the expression of breast cancer associated long noncoding RNA HOTAIR in vitro and in vivo. J. Steroid Biochem. Mol. Biol..

[66] Divoux A., Karastergiou K., Xie H., Guo W., Perera R.J., Fried S.K., Smith S.R. (2014). Identification of a novel lncRNA in gluteal adipose tissue and evidence for its positive effect on preadipocyte differentiation. Obesity.

[67] Meruvu S., Zhang J., Choudhury M. (2021). Butyl Benzyl Phthalate Promotes Adipogenesis in 3T3-L1 Cells via the miRNA-34a-5p Signaling Pathway in the Absence of Exogenous Adipogenic Stimuli. Chem. Res. Toxicol..

[68] Kim S.H., Park M.J. (2014). Phthalate exposure and childhood obesity. Ann. Pediatr. Endocrinol. Metab..

[69] Buser M.C., Murray H.E., Scinicariello F. (2014). Age and sex differences in childhood and adulthood obesity association with phthalates: Analyses of NHANES 2007–2010. Int. J. Hyg. Environ. Health.

[70] Zhang J., Choudhury M. (2021). Benzyl Butyl Phthalate Induced Early lncRNA H19 Regulation in C3H10T1/2 Stem Cell Line. Chem. Res. Toxicol..

[71] Darbre P., Darbre P. (2015). Endocrine Disruption and Human Health.

[72] Slotkin T.A. (2011). Does early-life exposure to organophosphate insecticides lead to prediabetes and obesity?. Reprod. Toxicol..

[73] Orton F., Rosivatz E., Scholze M., Kortenkamp A. (2011). Widely used pesticides with previously unknown endocrine activity revealed as in vitro antiandrogens. Environ. Health Perspect..

[74] Kirchner S., Kieu T., Chow C., Casey S., Blumberg B. (2010). Prenatal exposure to the environmental obesogen tributyltin predisposes multipotent stem cells to become adipocytes. Mol. Endocrinol..

[75] Janesick A., Blumberg B. (2011). Minireview: PPARgamma as the target of obesogens. J. Steroid Biochem. Mol. Biol..

[76] Chapman R.S., He X., Blair A.E., Lan Q. (2005). Improvement in household stoves and risk of chronic obstructive pulmonary disease in Xuanwei, China: Retrospective cohort study. BMJ.

[77] Hnizdo E., Sullivan P.A., Bang K.M., Wagner G. (2004). Airflow obstruction attributable to work in industry and occupation among U.S. race/ethnic groups: A study of NHANES III data. Am. J. Ind. Med..

[78] Hiraiwa K., van Eeden S.F. (2013). Contribution of lung macrophages to the inflammatory responses induced by exposure to air pollutants. Mediat. Inflamm..

[79] Yan Z., Zhang H., Maher C., Arteaga-Solis E., Champagne F.A., Wu L., McDonald J.D., Yan B., Schwartz G.J., Miller R.L. (2014). Prenatal polycyclic aromatic hydrocarbon, adiposity, peroxisome proliferator-activated receptor (PPAR) gamma methylation in offspring, grand-offspring mice. PLoS ONE.

[80] Kamstra J.H., Hruba E., Blumberg B., Janesick A., Mandrup S., Hamers T., Legler J. (2014). Transcriptional and epigenetic mechanisms underlying enhanced in vitro adipocyte differentiation by the brominated flame retardant BDE-47. Environ. Sci. Technol..

[81] Halden R.U. (2010). Plastics and health risks. Annu. Rev. Public Health.

[82] Reddivari L., Veeramachaneni D.N.R., Walters W.A., Lozupone C., Palmer J., Hewage M.K.K., Bhatnagar R., Amir A., Kennett M.J., Knight R. (2017). Perinatal Bisphenol A Exposure Induces Chronic Inflammation in Rabbit Offspring via Modulation of Gut Bacteria and Their Metabolites. mSystems.

[83] Lakind J.S., Goodman M., Mattison D.R. (2014). Bisphenol A and indicators of obesity, glucose metabolism/type 2 diabetes and cardiovascular disease: A systematic review of epidemiologic research. Crit. Rev. Toxicol..

[84] Ranciere F., Lyons J.G., Loh V.H., Botton J., Galloway T., Wang T., Shaw J.E., Magliano D.J. (2015). Bisphenol A and the risk of cardiometabolic disorders: A systematic review with meta-analysis of the epidemiological evidence. Environ. Health.

[85] Bonde J.P., Flachs E.M., Rimborg S., Glazer C.H., Giwercman A., Ramlau-Hansen C.H., Hougaard K.S., Hoyer B.B., Haervig K.K., Petersen S.B. (2016). The epidemiologic evidence linking prenatal and postnatal exposure to endocrine disrupting chemicals with male reproductive disorders: A systematic review and meta-analysis. Hum. Reprod. Update.

[86] Jalal N., Surendranath A.R., Pathak J.L., Yu S., Chung C.Y. (2018). Bisphenol A (BPA) the mighty and the mutagenic. Toxicol. Rep..

[87] Richter C.A., Birnbaum L.S., Farabollini F., Newbold R.R., Rubin B.S., Talsness C.E., Vandenbergh J.G., Walser-Kuntz D.R., vom Saal F.S. (2007). In vivo effects of bisphenol A in laboratory rodent studies. Reprod. Toxicol..

[88] Safe S.H., Pallaroni L., Yoon K., Gaido K., Ross S., McDonnell D. (2002). Problems for risk assessment of endocrine-active estrogenic compounds. Environ. Health Perspect..

[89] Hayes L., Weening A., Morey L.M. (2016). Differential Effects of Estradiol and Bisphenol A on SET8 and SIRT1 Expression in Ovarian Cancer Cells. Dose Response.

[90] Rubin B.S. (2011). Bisphenol A: An endocrine disruptor with widespread exposure and multiple effects. J. Steroid Biochem. Mol. Biol..

[91] Ben-Jonathan N., Hugo E.R., Brandebourg T.D. (2009). Effects of bisphenol A on adipokine release from human adipose tissue: Implications for the metabolic syndrome. Mol. Cell. Endocrinol..

[92] Carwile J.L., Michels K.B. (2011). Urinary bisphenol A and obesity: NHANES 2003-2006. Environ. Res..

[93] Menale C., Grandone A., Nicolucci C., Cirillo G., Crispi S., Di Sessa A., Marzuillo P., Rossi S., Mita D.G., Perrone L. (2017). Bisphenol A is associated with insulin resistance and modulates adiponectin and resistin gene expression in obese children. Pediatr. Obes..

[94] Codoner-Franch P., Alonso-Iglesias E. (2015). Resistin: Insulin resistance to malignancy. Clin. Chim. Acta.

[95] Wang J., Sun B., Hou M., Pan X., Li X. (2013). The environmental obesogen bisphenol A promotes adipogenesis by increasing the amount of 11beta-hydroxysteroid dehydrogenase type 1 in the adipose tissue of children. Int. J. Obes..

[96] Boucher J.G., Boudreau A., Atlas E. (2014). Bisphenol A induces differentiation of human preadipocytes in the absence of glucocorticoid and is inhibited by an estrogen-receptor antagonist. Nutr. Diabetes.

[97] Hatch E.E., Troisi R., Palmer J.R., Wise L.A., Titus L., Strohsnitter W.C., Ricker W., Hyer M., Hoover R.N. (2015). Prenatal diethylstilbestrol exposure and risk of obesity in adult women. J. Dev. Orig. Health Dis..

[98] Sun L., Goff L.A., Trapnell C., Alexander R., Lo K.A., Hacisuleyman E., Sauvageau M., Tazon-Vega B., Kelley D.R., Hendrickson D.G. (2013). Long noncoding RNAs regulate adipogenesis. Proc. Natl. Acad. Sci. USA.

[99] Cheng Y., Gao W.W., Tang H.M., Deng J.J., Wong C.M., Chan C.P., Jin D.Y. (2016). beta-TrCP-mediated ubiquitination and degradation of liver-enriched transcription factor CREB-H. Sci. Rep..

[100] Yang L., Li P., Yang W., Ruan X., Kiesewetter K., Zhu J., Cao H. (2016). Integrative Transcriptome Analyses of Metabolic Responses in Mice Define Pivotal LncRNA Metabolic Regulators. Cell Metab..

[101] Hajjari M., Salavaty A. (2015). HOTAIR: An oncogenic long non-coding RNA in different cancers. Cancer Biol. Med..

[102] Calafat A.M., McKee R.H. (2006). Integrating biomonitoring exposure data into the risk assessment process: Phthalates [diethyl phthalate and di(2-ethylhexyl) phthalate] as a case study. Environ. Health Perspect..

[103] Kelly D.M., Jones T.H. (2015). Testosterone and obesity. Obes. Rev..

[104] Svechnikov K., Izzo G., Landreh L., Weisser J., Soder O. (2010). Endocrine disruptors and Leydig cell function. J. Biomed. Biotechnol..

[105] Pereira-Fernandes A., Demaegdt H., Vandermeiren K., Hectors T.L., Jorens P.G., Blust R., Vanparys C. (2013). Evaluation of a screening system for obesogenic compounds: Screening of endocrine disrupting compounds and evaluation of the PPAR dependency of the effect. PLoS ONE.

[106] Pereira-Fernandes A., Vanparys C., Vergauwen L., Knapen D., Jorens P.G., Blust R. (2014). Toxicogenomics in the 3T3-L1 cell line, a new approach for screening of obesogenic compounds. Toxicol. Sci..

[107] Mangum L.H., Howell G.E., Chambers J.E. (2015). Exposure to p,p’-DDE enhances differentiation of 3T3-L1 preadipocytes in a model of sub-optimal differentiation. Toxicol. Lett..

[108] Peris-Sampedro F., Basaure P., Reverte I., Cabre M., Domingo J.L., Colomina M.T. (2015). Chronic exposure to chlorpyrifos triggered body weight increase and memory impairment depending on human apoE polymorphisms in a targeted replacement mouse model. Physiol. Behav..

[109] Traboulsi H., Guerrina N., Iu M., Maysinger D., Ariya P., Baglole C.J. (2017). Inhaled Pollutants: The Molecular Scene behind Respiratory and Systemic Diseases Associated with Ultrafine Particulate Matter. Int. J. Mol. Sci..

[110] Bevan G.H., Al-Kindi S.G., Brook R., Rajagopalan S. (2021). Ambient Air Pollution and Atherosclerosis: Recent Updates. Curr. Atheroscler. Rep..

[111] Samet J.M., Dominici F., Curriero F.C., Coursac I., Zeger S.L. (2000). Fine particulate air pollution and mortality in 20 U.S. cities, 1987–1994. N. Engl. J. Med..

[112] Vineis P., Husgafvel-Pursiainen K. (2005). Air pollution and cancer: Biomarker studies in human populations. Carcinogenesis.

[113] Bjorntorp P., Rosmond R. (2000). Obesity and cortisol. Nutrition.

[114] Harris R.B. (2015). Chronic and acute effects of stress on energy balance: Are there appropriate animal models?. Am. J. Physiol. Regul. Integr. Comp. Physiol..

[115] Elobeid M.A., Padilla M.A., Brock D.W., Ruden D.M., Allison D.B. (2010). Endocrine disruptors and obesity: An examination of selected persistent organic pollutants in the NHANES 1999-2002 data. Int. J. Environ. Res. Public Health.

[116] Turyk M.E., Anderson H.A., Steenport D., Buelow C., Imm P., Knobeloch L. (2010). Longitudinal biomonitoring for polybrominated diphenyl ethers (PBDEs) in residents of the Great Lakes basin. Chemosphere.

[117] Dirinck E., Jorens P.G., Covaci A., Geens T., Roosens L., Neels H., Mertens I., Van Gaal L. (2011). Obesity and persistent organic pollutants: Possible obesogenic effect of organochlorine pesticides and polychlorinated biphenyls. Obesity.

[118] Dhooge W., Den Hond E., Koppen G., Bruckers L., Nelen V., Van De Mieroop E., Bilau M., Croes K., Baeyens W., Schoeters G. (2010). Internal exposure to pollutants and body size in Flemish adolescents and adults: Associations and dose-response relationships. Environ. Int..

[119] Skinner M.K., Manikkam M., Guerrero-Bosagna C. (2010). Epigenetic transgenerational actions of environmental factors in disease etiology. Trends Endocrinol. Metab..

[120] Anway M.D., Cupp A.S., Uzumcu M., Skinner M.K. (2005). Epigenetic transgenerational actions of endocrine disruptors and male fertility. Science.

[121] Manikkam M., Tracey R., Guerrero-Bosagna C., Skinner M.K. (2013). Plastics derived endocrine disruptors (BPA, DEHP and DBP) induce epigenetic transgenerational inheritance of obesity, reproductive disease and sperm epimutations. PLoS ONE.

[122] Manikkam M., Tracey R., Guerrero-Bosagna C., Skinner M.K. (2012). Pesticide and insect repellent mixture (permethrin and DEET) induces epigenetic transgenerational inheritance of disease and sperm epimutations. Reprod. Toxicol..

[123] Manikkam M., Tracey R., Guerrero-Bosagna C., Skinner M.K. (2012). Dioxin (TCDD) induces epigenetic transgenerational inheritance of adult onset disease and sperm epimutations. PLoS ONE.

[124] Tracey R., Manikkam M., Guerrero-Bosagna C., Skinner M.K. (2013). Hydrocarbons (jet fuel JP-8) induce epigenetic transgenerational inheritance of obesity, reproductive disease and sperm epimutations. Reprod. Toxicol..

[125] Skinner M.K., Manikkam M., Tracey R., Guerrero-Bosagna C., Haque M., Nilsson E.E. (2013). Ancestral dichlorodiphenyltrichloroethane (DDT) exposure promotes epigenetic transgenerational inheritance of obesity. BMC Med..

[126] Skinner M.K. (2011). Environmental epigenetic transgenerational inheritance and somatic epigenetic mitotic stability. Epigenetics.

[127] Skinner M.K., Manikkam M., Haque M.M., Zhang B., Savenkova M.I. (2012). Epigenetic transgenerational inheritance of somatic transcriptomes and epigenetic control regions. Genome Biol..

[128] Choi Y.J., Lee Y.A., Hong Y.C., Cho J., Lee K.S., Shin C.H., Kim B.N., Kim J.I., Park S.J., Bisgaard H. (2020). Effect of prenatal bisphenol A exposure on early childhood body mass index through epigenetic influence on the insulin-like growth factor 2 receptor (IGF2R) gene. Environ. Int..

[129] Manikkam M., Haque M.M., Guerrero-Bosagna C., Nilsson E.E., Skinner M.K. (2014). Pesticide methoxychlor promotes the epigenetic transgenerational inheritance of adult-onset disease through the female germline. PLoS ONE.

[130] Ozgyin L., Erdos E., Bojcsuk D., Balint B.L. (2015). Nuclear receptors in transgenerational epigenetic inheritance. Prog. Biophys. Mol. Biol..

[131] Bhan A., Hussain I., Ansari K.I., Bobzean S.A., Perrotti L.I., Mandal S.S. (2014). Histone methyltransferase EZH2 is transcriptionally induced by estradiol as well as estrogenic endocrine disruptors bisphenol-A and diethylstilbestrol. J. Mol. Biol..

[132] Doherty L.F., Bromer J.G., Zhou Y., Aldad T.S., Taylor H.S. (2010). In utero exposure to diethylstilbestrol (DES) or bisphenol-A (BPA) increases EZH2 expression in the mammary gland: An epigenetic mechanism linking endocrine disruptors to breast cancer. Horm. Cancer.

[133] Lee D.H., Jacobs D.R., Porta M. (2009). Hypothesis: A unifying mechanism for nutrition and chemicals as lifelong modulators of DNA hypomethylation. Environ. Health Perspect..

[134] Godfrey K.M., Sheppard A., Gluckman P.D., Lillycrop K.A., Burdge G.C., McLean C., Rodford J., Slater-Jefferies J.L., Garratt E., Crozier S.R. (2011). Epigenetic gene promoter methylation at birth is associated with child’s later adiposity. Diabetes.

